# Supplementary material for: Ulcerative Colitis and Acute Severe Ulcerative Colitis Patients Are Overlooked in Infliximab Population Pharmacokinetic Models: Results from a Comprehensive Review
Source: Pharmaceutics. 2022 Sep 30;14(10):2095. doi: 10.3390/pharmaceutics14102095 (PMC9610912; doi:10.3390/pharmaceutics14102095)
Supplement: Supplementary file 1 [file pharmaceutics-14-02095-s001.zip › pharmaceutics-1869725-Supplementary Figure S1.pdf]

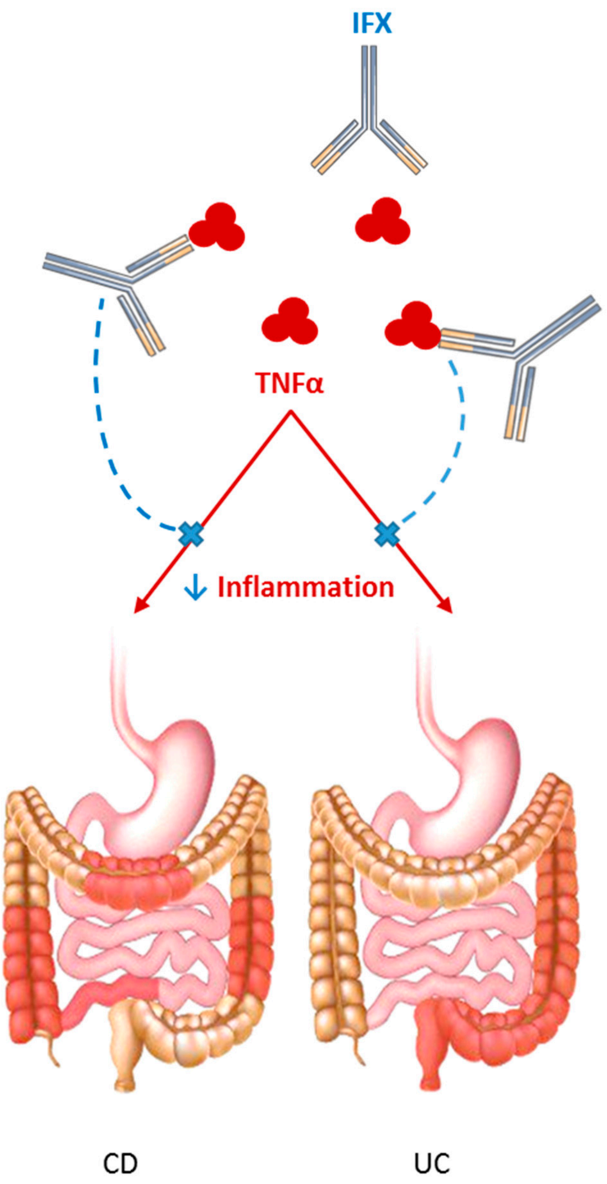

Figure S1. Scheme of IFX pharmacological action on TNFα.
Red lines: inflammatory effect; blue lines and cross: inhibition effect of IFX on TNFα.
Abbreviations: CD: Crohn's disease; IFX: Infliximab; TNFα: Tumor necrosis factor α; UC: Ulcerative colitis.
