# Supplementary material for: Ulcerative Colitis and Acute Severe Ulcerative Colitis Patients Are Overlooked in Infliximab Population Pharmacokinetic Models: Results from a Comprehensive Review
Source: Pharmaceutics. 2022 Sep 30;14(10):2095. doi: 10.3390/pharmaceutics14102095 (PMC9610912; doi:10.3390/pharmaceutics14102095)
Supplement: Supplementary file 1 [file pharmaceutics-14-02095-s001.zip › pharmaceutics-1869725-Supplemnetary Table S1.pdf]

Supplematary information

Table S1. Selected Population pharmacokinetic models for infliximab in ulcerative colitis/inflammatory bowel diseases patients.

**Model**	**Structural PK model**				**Covariate model**	**IIV/IOV model**	**Residual error model**	**Specified implementation**			
	Parameter estimates (RSE)				*Parameter estimate (RSE)*	*CV (RSE)*	*Type: parameter estimate (RSE)*	*Full model code*	*ODEs/ model schematic*	*Covariate relationships*	*Random effects*
**Ternant-model (2008)**	**2-CMT model, first order elimination**							NO	NO	YES	YES
Article	*CL (L/h)*	*V1 (L)*	*V2 (L)*	*Q (L/h)*							
Ternant (2008)[1]	ADA-: 0.012 (7.9%)	Smen: 2.3 (18.1%)	1.0 (22.6%)	0.0054 (34.5)	NA, see structural model		Additiative: 1.04 mg/L (12.0%)				
	ADA+: 0.032 (10.9%)	women: 1.1 (31.4%)				*CL ADA-*: 22.5%					
		WT: 1.7 (19.6%)				*CL ADA+*: 22.7%					
						$V1_{men}$: 14.1%					
						$V1_{women}$: 11.3%					
						V1 WT: 1.719.3%					
						V2: 15%					
						Q: 10%					
**Fasanmade-model (2009)**	**2-CMT model, first order elimination**							NO	NO	YES	YES
Article	*CL (L/day)*	*V1 (L)*	*V2 (L)*	*Q (L/day)*							
Fasanmade (2009)[2]	0.407 L/day (2.5%)	3.29 L (2.1%)	4.13 L (3.9%)	7.14 L/day (6.8%)		CV (RSE)	Proportional error: 40.30% (2.6%)				
					ALB~CL: -1.54 (2.5%)	*CL*: 37.68% (8.5%)	Additive error: 0.0413 µg/L (3.5%)				

					ADA~CL: 0.471 (22.5%)<br>SEX_{Female}~CL: -0.236 (11.9%)<br>WT~V1: 0.538 (13.5%)<br>SEX_{Female}~V1: -0.137 (23.2%)	*V1*: 22.11 (16.6%)					
Vande Casteele (2019)[3]	*Fasanmade (2009)*				*Fasanmade (2009)*	*Fasanmade (2009)*	*Fasanmade (2009)*	*NO*	*NO*		
Kimura (2019)[4]	*Fasanmade (2009, 2011)*				*Fasanmade (2009, 2011)*	*Fasanmade (2009, 2011)*	*Fasanmade (2009, 2011)*	*NO*	*YES*		
Kimura (2020)[5]	CD: 0.353 (0.212–0.442)<br>UC: 0.450 (0.223–0.811)	CD: 2.97 (1.96–5.00)<br>UC: 2.25 (1.60–3.22)	CD: 0.137 (0.0944–0.273)<br>UC: 3.71 (3.29–4.06)	CD: 1.22 (1.10–1.51)<br>UC 7.10 (6.96–7.32)	*NA*	*NA*	*NA*	*NO*	*NO*		
**Fasanmade-model (2011, CD)**	**2-CMT model, first order elimination**							NO	NO	YES	YES
Article	*CL (mL/kg/day)*	*V1 (mL/kg)*	*V2 (mL/kg)*	*Q (mL/kg/day)*							
Fasanmade (2011)[6]	5.42 (2.0)	52.4 (0.9)	19.6 (4.2)	2.26 (9.9)	ALB~CL: –0.855 (13.5%)<br>ADA~CL: 0.291 (15.2%)	CL: 30.7% (8.1%)<br>V1: 12.6% (19.2%)	Proportional error: 29.2% (2.7)<br>Additive error: 0.371 µg/mL (18.3%)				
					IMM~CL: –0.137 (20.5%)<br>WT~CL: –0.313 (14.6%)<br>WT~V1: –0.233 (12.3%)	V2: 55.3% (18.5%)					

					WT~V2: –0.588 (16.1%)						
Santacana (2018)[7]	*Fasanmade (2011)*				*Fasanmade (2011)*	*Fasanmade (2011)*	*Fasanmade (2011)*	*NO*	*NO*		
Kimura (2019)	*see above under Fasanmade (2009)*										
Kimura (2020)	*see above under Fasanmade (2009)*										
Candel (2020)[8]	*kel (1/h)*	*V1 (L)*	*V2 (L)*	*Q (L/h)*				NO	NO		
	0.0038 (SD: 0.0011)	3.57 L (SD: 0.041)	1.43 L (SD: 0.39)	0.014 L/h (SD: 0.004)	*Fasanmade (2011)*	*Fasanmade (2011)*	*Fasanmade (2011)*				
Santacana (2020)[9]	*Fasanmade (2011)*				*Fasanmade (2011)*	*Fasanmade (2011)*	*Fasanmade (2011)*	*NO*	*NO*		
Bauman (2020)[10]	*Fasanmade (2011)*				$CL_{ind} = CL_{pop} \times (WT/65)^{0.7} \times (ALB/3.5)^{-1.1} \times (ESR/9)^{0.11} \times 1.18^{ADAlevel}$	NS	NA	NO	NO		
**Xu-model (2012)**	**2-CMT model, first order elimination**							NO	NO	YES	YES
Article	*CL (L/h)*	*V1 (L)*	*V2 (L)*	*Q (mL/kg/day)*							
Xu (2012)[11]	0.296 (1.5%)	3.3 (1.1%)	1.16 (4.9%)	0.0781 (11.4%)	WT~CL : 0.612 (4.7%)<br>ALB~CL: -2.3 (9.3%)<br>ADA~CL: 0.231 (24.7%)<br>WT~V1: 0.696 (2.9%)<br>WT~V2: 0.604 (19.5%)<br>WT~Q: 1.15 (21.2%)	CL: 31.3% (6.9%)<br>V1: 9.85% (33.9%)<br>V2: 76.1% (12.3%)<br>Q: 111% (14.4%)	Proportional: 41.9% (1.0%)				

Dubinsky (2017)[11]	Xu (2012)				Xu (2012)	Xu (2012)	Xu (2012)	NO	NO
Wojciechowski (2017) [12]	Xu (2012)				Xu (2012)	Xu (2012)	Xu (2012)	NO	
Eser (2018)[13]								NO	NO
Model 1	0.246 (10.1%)	4 (20.1%)	2.34 (28.8%)	0.07 (20.5%)	WT~CL: 1.59 (20.1%)<br>ALB~CL: -1.45 (27.7%)<br>ADA~CL: 0.44 (23%)<br>WT~V1: 2.16 (28.6%)<br>WT~V2: NE<br>WT~Q: NE	CL: 33.02% (33.1%)<br>V1: 40.12% (58.7%)<br>V2: 122.07% (40.3%)<br>Q: NE	Proportional: 53.10% (5.1%)		
Model 2	0.28 (NE)	3.16 (NE)	0.477 (NE)	0.04 (NE)	WT~CL: 1.56 (NE)<br>ALB~CL: -0.763 (NE)<br>ADA~CL: 0.76 (NE)<br>WT~V1: 2.25 (NE)<br>WT~V2: NE<br>WT~Q: NE	CL: 24.04% (NE)<br>V1: 56.66% (NE)<br>V2: 81.30% (NE)<br>Q: NE	Proportional: 54.40% (NE)		
Model 3	0.243 (7.7%)	4.04 (13.2%)	3.02 (52.3%)	0.03 (24.4%)	WT~CL: 0.921 (29%)<br>ALB~CL: -1.24 (28.1%)<br>ADA~CL: 1.11 (13.5%)<br>WT~V1: 1.12(41.3%)<br>WT~V2: NE<br>WT~Q: NE	CL:25.10% (47.5%)<br>V1: 49.80% (32.6%)<br>V2: 92.47% (68.1%)<br>Q: NE	Proportional: 56.70% (4.7%)		

Dubinsky (2022)[14]	Xu (2012)				Xu (2012)	Xu (2012)	Xu (2012)	NO	NO		
**Dotan-model (2014)**	**2-CMT model, first order elimination**							NO	NO	YES	YES
Article	*CL (L/day)*	*V1 (L)*	*V2 (L)*	*Q (L/day)*							
Dotan (2014)[15]	0.381 (0.5%)	2.37 (0.2%)	1.37 (0.4%)	0.122 (0.4%)	WT~CL: 0.612 (2.1%)<br>ALB~CL: -1.39 (1.5%)<br>ADA~CL: 1.59 (13%)<br>WT~V1: 0.696 (1.9%)<br>WT~Q: 1.15 (3.6%)<br>WT~V2: 0.604 (10.2%)	CL: 13.45 (4%)<br>V1: 42.07 (1.5%)<br>V2: 32.40 (1.7%)<br>Q: 85.15 (1.2%)	NR				
**Buurman-model (2015)**	**2-CMT model, first order elimination**							NO	NO	YES	NO
Article	*CL (L/day)*	*V1 (L)*	*V2 (L)*	*Q (L/day)*							
Buurman (2015)[16]	0.199 (6%)	4.94 (10%)	3.13 (32%)	0.0618 (23%)	Period~CL: +40% (11%)<br>ADA~CL: +72% (35%)<br>SEX_{male}~CL: +35% (34%)<br>HBI~V1: -3.6% (28%)	CL: 18% (18%)<br>V1: 17.1% (31%)	Proportional: 21.7% (30%)<br>Additional: 0.98 mg/L (18%)				
**Passot-model (2016)**	**1-CMT model, first order elimination**							NO	NO	YES	YES
Article	*CL (L/day)*	*V1 (L)*									
Passot (2016)[17]	0.23 (4%)	5.2 (4%)			SEX_{Male}~V: 0.209 (45%)<br>$Age_{<=15}$~V: -0-396 (37%	V: 0.224 (12%)<br>CL: 0.304 (7%)	Proportional: 0.223 (7%)<br>Additional: 0.72 mg/L (19%)				

					CD~V: 0.399 (17%)<br>WT~CL: 0.603 (18%)<br>SEX_{Male}~CL: 0.181 (30%)<br>CD on CL: 0.384 (15%)<br>UC~CL: 0.472 (21%)<br>RA~CL: 0.392 (32%)<br>MTX~CL: -0.336 (52%)						
**Brandse-model (2017)**	**2-CMT model, first order elimination**							NO	N	NO	NO
Article	*CL (L/day)*	*V1 (L)*	*V2 (L)*	*Q (L/day)*							
Brandse (2017)[18]	0.358	4.72	2.4	0.0697	WT~CL: 0.523<br>ALB~CL: -1.38<br>Previous exposure ~CL: 0.0521<br>ADA~CL: 0.601<br>WT~V_c: 0.473<br>WT~Q: 0.523<br>WT~V_p: 0.473	CL: 38.1%<br>V1: 68.6%<br>V2: 71.7%<br>Q: 58.1%	NR				
**Kevans-model (2018)**	**2-CMT model, time varying CL**							NO	NO	NO	NO
Article	*CL (L/day)*	*V1 (L)*	*V2 (L)*	*Q (L/day)*							
Kevans (2018)[19]	0.368 (0.5%)	3.3 (0.2%)	3.42 (0.5%)	0.308 (0.1%)	rate constant of CL increase with ADA: 0.138 (25.5%)	CL: 50.10% (1.2%)	Proportional error: 28.5% (0.3%)				

	Time dependent CL: 0.105 (0.4%)					WT~CL: 0.709 (0%)<br>ALB~CL: -0.445 (2.6%)<br>ADA~CL: -0.0373 (4.9%)<br>WT~V1: 0.64 (0%)<br>WT~V2: 0.991 (0%)<br>WT~Q: 1.52 (0%)	V1: 35.92% (0.8%)<br>V2: 75.70% (0.8%)<br>Q: 55.14% (0.4%)					
**Petitcollin-model (2019)**	**1-CMT model, time varying CL**			**logit-risk model of immunization**					NO	NO	YES	YES
Article	*CL_base (L/day)*	Slope (L/day/year)	*V1 (L)*	CLvar 1 FIX		UC~CL: 0.377 (29%)	V: 25.4% (15%)	Proportional: 20.6% (6%)				
Petitcollin (2019)[20]	0.273 (7%)	0.0348 (9%)	11.5 (5%)	intercept: 10.9 (5%)<br>Beta: 0.0526 (21%)		Dose~CL: −0.267 (22%)<br>CRP~CL: 0.0654 (15%)<br>Mayo score~CL: 0.0934 (30%)<br>AZA~CL: 0.849 (2%)<br>WT~Slope: 31.1 (31%)	CL_{base}: 44.3% (10%)<br>β_{dose}: 33.3% (19%)<br>slope: 32% (26%)<br>beta: 102% (17%)<br>CL_{var}: 44.4% (11%)	Additional: 0.446 ug/mL (22%)				
**Berends-model (2019)**	**2-CMT model, first order elimination**				**TMDD model**				YES	YES	YES	YES
Article	*CL (L/day)*	*V1 (L)*	*V2 (L)*	*Q (L/day)*								
Berends (2019)[21]	0.404 (9.9%)	3.18 (9.1%)	1.64 (6.3%)	0.344 (20%)	Bmax : 0.38 pM (20%)<br>Bmax : 19.8 pg/mL (-)<br>Kss : 14 nM (24%)	ADA~CL: 2.15 (12%)<br>ALB~CL: -1.13 (36%)	CL: 29.2% (19%)<br>V1: 22.7% (16%)<br>V2: 74.2% (19%)	Proportional IFX: 0.210 (13%)<br>Proportional TNF: 0.406 (9%)				

					Ke : 0.984 1/d (19%)<br><br>Kdeg : 5.12 1/d (-)			CL-V1: 12.3% (106%)<br>Bmax: 39.4% (16%)					
**Dreesen-model (2019)**	**1-CMT model, first order elimination**									YES	YES	YES	YES
Article	*ke (1/day)*	*V (L)*											
Dreesen (2019)[22]	MAYO 1: 0.0521 (11%)<br>MAYO 2: 0.0543 (3.6%)<br>MAYO 3: 0.0667 (8.1%)	6.34 (3.2%)					CRP~Ke: 0.0859 (25%)<br><br>ALB~Ke: -0.808 (39%)<br><br>FFM~V: 0.544 (29%)<br><br>Corticosteroid ~V: 1.33 (6.9%)<br>Pancolitis ~V: 1.23 (0.019%)	Ke: 29.8% (7.8%)<br><br>V: 26.5% (20%)<br><br>IOV Ke: 18.7% (17%)	Proportional: 19.2 % (9.4%)<br><br>Additional: 0.300 ug/mL (FIX)				
Faelens (2021)[23]	MAYO 1: 0.0422 (14%)<br>MAYO 2: 0.0463 (3.8%)<br>MAYO 3: 0.0570 (5.0%)	6.97 (5.0%)					CRP~Ke: Not included<br><br>ALB~Ke: Not included<br><br>FFM~V: 0.517 (39%)<br><br>Corticosteroid ~V: 1.30 (7.7%)<br>Pancolitis ~V: 1.25 (6.9%)	Ke: 33.4% (5.78%)<br><br>V: 23.6% (41%)<br><br>IOV Ke: 6.7% (100%)	Proportional: 32.9 % (5.8%)<br><br>Additional: 0.300 ug/mL (FIX)	YES	NO		
**Hanzel-model (2021)**	**2-CMT model, first order elimination, subcutaneous admin.**									NO	YES	YES	YES
Article	*CL (L/day)*	*V1 (L)*	*V2 (L)*	*Q (L/day)*	*ka (1/day)*	*F (%)*							

Hanzel (2021)[24]	0.355 (2%)	3.10 (3%)	1.93 (2%)	0.598 (4%)	0.273 (8%)	79.1 (3%)	ALB~CL: -0.826 (11%)<br>BW~CL: 0.666 (15%)<br>ADA~CL: 1,39 (3%)<br>BW~V1: 0.385 (34%)<br>BW~V2: 1.08 (9%)<br>BW~Q: 1.26 (15%)	CL: 27.7% (14%)<br>V1: 21.4% (21%)<br>ka 48.5% (45%)<br>F1: 16.4% (15%)<br>CL-V1:0.028% (31.4%)<br>CL-ka: -0.046% (53.2%)<br>CL-F1: -0.013% (42.2%)<br>V1-ka: -0.069% (47%)<br>V1-F1: 0.00008% (744%)<br>ka-F1: 0.003% (413%)<br>IOV CL: 17.5% (6%)	Proportional: 0.102 (2%)<br>Additional: 1.66 (3%) ug/mL				
**Grisic-model (2021)**	**2-CMT model, first order elimination**									NO	YES	YES	YES
	CL (L/h)	*V1 (L)*	*V2 (L)*	*Q (L/h)*									
Grisic (2021)[25]	0.0109 (3%)	3.67 (-)	0.956 (11%)	0.0067 (-)			ALB~CL: -1.17 (21%)<br>ADA~CL: 0.972 (4%)<br>IMM~CL: 0.847 (5%)<br>WT~CL:0.356 (41%)	CL: 34.9% (8%)<br>V1: 12.8% (-)<br>V2: 55.3% (-)	Proportional error: 24% (14%)<br>Additive error (SD): 0.478 (21%) μg/mL				

ADA: Anti-drug antibodies; ADA_{cat}: Categorical ADA; Add: additional residual variability; ALB: Albumin concentrations; AZA: Azathioprine; B_{maxTNF}: Baseline TNF concen-tration; BW: Body weight; CD: Crohn's disease; CL: Clearance; CL_{base}: Baseline clearance; CL_{time}: Time dependant CL; CMT: Compartment; CORT: Corticosteroids; CRP: C-Reactive protein; F: Bioavailability; FFM: Free fat mass; HBI: Harvey-Bradshaw index; IFX: Infliximab; IIV: Interindividual variability; IMM: Immunomodulators; IOV: Interoccasion variability; ka: Absorption rate constant; $K_{ss,TNF-IFX}$: Steady-state equilibrium constant; $k_{deg,TNF}$: Degradation constant TNF receptor; ke: elimination rate con-stant; ke(p),TNF-IFX: Internalisation rate complex; MTX: Methotrexate; NR: Not reported; ODE: Ordinary differential equation; Prop: Proportional residual variability; Q: Inter-compartmental clearance; V1: Central volume of distribution; V2: Peripheral volume of distribution; RA: Rheumatoid arthritis; s.c.: subcutaneous; SD: Standard deviation; TMDD: Target mediated drug disposition; TNF: Tumour necrosis factor; UC: Ulcerative colitis; WT: Weight; y.o.: Years old.

References

1. Ternant, D. *et al.* Infliximab Pharmacokinetics in Inflammatory Bowel Disease Patients. *Therapeutic Drug Monitoring* **30**, (2008).
2. Fasanmade, A. A. *et al.* Population pharmacokinetic analysis of infliximab in patients with ulcerative colitis. *European Journal of Clinical Pharmacology* **65**, 1211–1228 (2009).
3. vande Casteele, N., Jeyarajah, J., Jairath, V., Feagan, B. G. & Sandborn, W. J. Infliximab Exposure-Response Relationship and Thresholds Associated With Endoscopic Healing in Patients With Ulcerative Colitis. *Clinical Gastroenterology and Hepatology* **17**, 1814-1821.e1 (2019).
4. Kimura, K., Yoshida, A., Katagiri, F., Takayanagi, R. & Yamada, Y. Prediction of clinical effects of infliximab administered for inflammatory bowel disease based on pharmacokinetic and pharmacodynamic modeling. *Biopharmaceutics & Drug Disposition* **40**, 250–261 (2019).
5. Kimura, K., Yoshida, A., Katagiri, F., Takayanagi, R. & Yamada, Y. Prediction of treatment failure during infliximab induction therapy in inflammatory bowel disease patients based on pharmacokinetic and pharmacodynamic modeling. *European Journal of Pharmaceutical Sciences* **150**, 105317 (2020).
6. Fasanmade, A. A., Adedokun, O. J., Blank, M., Zhou, H. & Davis, H. M. Pharmacokinetic Properties of Infliximab in Children and Adults with Crohn's Disease: A Retrospective Analysis of Data from 2 Phase III Clinical Trials. *Clinical Therapeutics* **33**, 946–964 (2011).
7. Santacana, E. *et al.* External evaluation of population pharmacokinetic models of infliximab in patients with inflammatory bowel disease. *Therapeutic Drug Monitoring* **40**, 120–129 (2018).

8. Gil Candel, M. *et al.* Usefulness of population pharmacokinetics to optimize the dosage regimen of infliximab in inflammatory bowel disease patients. *Revista espanola de enfermedades digestivas : organo oficial de la Sociedad Espanola de Patologia Digestiva* **112**, 590–597 (2020).

9. Santacana Juncosa, E. *et al.* Bayes-based dosing of infliximab in inflammatory bowel diseases: Short-term efficacy. *British Journal of Clinical Pharmacology* 1–12 (2020) doi:10.1111/bcp.14410.

10. Bauman, L. E. *et al.* Improved Population Pharmacokinetic Model for Predicting Optimized Infliximab Exposure in Pediatric Inflammatory Bowel Disease. *Inflammatory Bowel Diseases* **26**, 429–439 (2020).

11. Dubinsky, M. C., Phan, B. L., Singh, N., Rabizadeh, S. & Mould, D. R. Pharmacokinetic Dashboard-Recommended Dosing Is Different than Standard of Care Dosing in Infliximab-Treated Pediatric IBD Patients. *AAPS Journal* **19**, 215–222 (2017).

12. Wojciechowski, J., Upton, R. N., Mould, D. R., Wiese, M. D. & Foster, D. J. R. Infliximab Maintenance Dosing in Inflammatory Bowel Disease: an Example for In Silico Assessment of Adaptive Dosing Strategies. *The AAPS Journal* **19**, 1136–1147 (2017).

13. Eser, A. *et al.* Prediction of Individual Serum Infliximab Concentrations in Inflammatory Bowel Disease by a Bayesian Dashboard System. *Journal of Clinical Pharmacology* **58**, 790–802 (2018).

14. Dubinsky, M. C. *et al.* Dashboard-Driven Accelerated Infliximab Induction Dosing Increases Infliximab Durability and Reduces Immunogenicity. *Inflamm Bowel Dis* (2022) doi:10.1093/IBD/IZAB285.

15. Dotan, I. *et al.* Patient factors that increase infliximab clearance and shorten half-life in inflammatory bowel disease: A population pharmacokinetic study. *Inflammatory Bowel Diseases* **20**, 2247–2259 (2014).

16. Buurman, D. J., Maurer, J. M., Keizer, R. J., Kosterink, J. G. W. & Dijkstra, G. Population pharmacokinetics of infliximab in patients with inflammatory bowel disease: Potential implications for dosing in clinical practice. *Alimentary Pharmacology and Therapeutics* **42**, 529–539 (2015).

17. Passot, C. *et al.* The underlying inflammatory chronic disease influences infliximab pharmacokinetics. *MAbs* **8**, 1407–1416 (2016).

18. Brandse, J. F. *et al.* A Real-life Population Pharmacokinetic Study Reveals Factors Associated with Clearance and Immunogenicity of Infliximab in Inflammatory Bowel Disease. *Inflammatory Bowel Diseases* **23**, 650–660 (2017).

19. Kevans, D., Murthy, S., Mould, D. R. & Silverberg, M. S. Accelerated clearance of infliximab is associated with treatment failure in patients with corticosteroid-refractory acute ulcerative colitis. *Journal of Crohn's and Colitis* **12**, 662–669 (2018).

20. Petitcollin, A. *et al.* Pharmacokinetic Parameters of Infliximab Influence the Rate of Relapse After De-Escalation in Adults With Inflammatory Bowel Diseases. *Clinical Pharmacology and Therapeutics* **106**, 605–615 (2019).

21. Berends, S. E. *et al.* Tumor necrosis factor-mediated disposition of infliximab in ulcerative colitis patients. *Journal of Pharmacokinetics and Pharmacodynamics* **46**, 543 (2019).

22. Dreesen, E. *et al.* Optimising infliximab induction dosing for patients with ulcerative colitis. *British Journal of Clinical Pharmacology* **85**, 782–795 (2019).

23. Faelens, R. *et al.* Model-informed precision dosing during infliximab induction therapy reduces variability in exposure and endoscopic improvement between patients with ulcerative colitis. *Pharmaceutics* **13**, (2021).

24. Hanzel, J., Bukkems, L. H., Gecse, K. B., D'Haens, G. R. & Mathôt, R. A. A. Population pharmacokinetics of subcutaneous infliximab CT-P13 in Crohn's disease and ulcerative colitis. *Aliment Pharmacol Ther* **54**, 1309–1319 (2021).

25. Grisic, A. M., Eser, A., Huisinga, W., Reinisch, W. & Kloft, C. Quantitative relationship between infliximab exposure and inhibition of C-reactive protein synthesis to support inflammatory bowel disease management. *British Journal of Clinical Pharmacology* **87**, 2374–2384 (2021).
